# Management of hip fracture in older adults with cognitive impairment: a narrative review

**DOI:** 10.3389/fpubh.2026.1816268

**Published:** 2026-04-29

**Authors:** Hao Rao, Li Luo, Jialing Cheng, Gang Wu, Hongmei Zhang, Xuxing Zhang, Jian Qin, Zidong Wang

**Affiliations:** 1 First Clinical College, Hubei University of Chinese Medicine, Wuhan, Hubei, China; 2 Affiliated Hospital of Hubei University of Chinese Medicine, Hubei Provincial Hospital of Traditional Chinese Medicine, Hubei Provincial Institute of Traditional Chinese Medicine, Wuhan, Hubei, China

**Keywords:** cognitive dysfunction, delirium, geriatric co-management, hip fractures, neuropsychological tests, postoperative cognitive complications, surgery

## Abstract

**Background:**

Hip fractures in older adults are costly and often fatal. Cognitive impairment (CI) complicates pain assessment, raises delirium risk, and hinders early mobilization and rehabilitation. This review synthesizes recent evidence on postoperative care, covering epidemiology, mechanisms, delirium, rehab, outcomes, and practical bedside strategies.

**Methods:**

A narrative review was performed to inform postoperative management of older adults hip-fracture patients with CI. PubMed and Web of Science were searched from 2018 to 2025 using MeSH and free-text terms for hip fracture, cognitive dysfunction/dementia/MCI, and rehabilitation, delirium, perioperative care, orthogeriatrics, and outcomes. Only English-language human studies were included. CI was defined by DSM-5/ICD-10 dementia, Petersen MCI, or MMSE <24/MoCA <26. Two reviewers independently screened studies; disagreements were resolved by a senior researcher. Case reports, protocols, abstracts, animal studies, <50 participants, non-core topics, and short follow-up were excluded. Additional 2023–2025 studies were identified by targeted searches and reference tracing. No risk-of-bias or GRADE assessment was applied.

**Results:**

Among older adults with hip fracture, approximately 20–40% have CI, making it a prominent clinical characteristic of this population. Dementia and even mild cognitive deficits increase the risk of falls and hip fractures by two- to threefold. After fracture, CI is linked to worse outcomes, including more delirium and complications, slower recovery, and higher mortality. Delirium is among the most prominent postoperative complications, particularly in those with pre-existing CI. Multidisciplinary care models, such as orthogeriatric co-management, early surgery within 24 to 48 h, adequate analgesia, and proactive delirium prevention, are associated with better outcomes. Rehabilitation should not be considered futile in dementia, yet patients with CI often receive less therapy and are less likely to regain pre-fracture function. One-year mortality and institutionalization are higher, while education and cognitive reserve may buffer decline, highlighting CI heterogeneity.

**Conclusion:**

Hip fracture in older adults with CI represents a high-risk clinical scenario that often warrants coordinated interdisciplinary care. Current clinical recommendations are supported by a hierarchy of evidence ranging from Level I (meta-analyses and RCTs) for orthogeriatric co-management and analgesia to Level II (prospective cohorts) for long-term functional outcomes. Available evidence generally supports early surgery, geriatric co-management, delirium prevention, effective analgesia, early mobilization, and tailored rehabilitation. These strategies may support recovery and may be associated with lower mortality and less loss of independence, though gaps remain in optimal rehab intensity and long-term cognitive outcomes.

## Introduction

Hip fractures represent a major public health issue among older adults, with a one-year mortality rate of approximately 20–30% (1). In 2019, about 1.4 million hip fractures occurred globally, and this number is projected to exceed 5 million by 2050 due to the aging population (1). The prevalence of dementia and milder forms of cognitive impairment (CI) also increases with age. Approximately 20% of patients with hip fractures have diagnosed CI, while up to 40% may exhibit some degree of cognitive vulnerability when broader criteria are applied (2).

The coexistence of hip fracture and CI presents a complex clinical challenge. Cognitive disorders, particularly dementia, significantly increase the risk of falls and fractures (3), while the stress of fracture, surgery, and hospitalization may precipitate acute and persistent cognitive decline, such as delirium or long-term impairment (4). Effective postoperative management therefore requires coordinated, interdisciplinary care that addresses both the orthopedic injury and the patient’s cognitive and functional needs. Emerging data suggest that surgery-induced systemic inflammation may contribute to postoperative neurocognitive disorders, offering a potential intervention target. Mounting evidence indicates that perioperative inflammatory cascades may interact with underlying neurodegenerative pathways, thereby accelerating neuronal dysfunction and synaptic impairment in vulnerable older adults. Recent studies have further linked neurodegenerative processes, iron dysregulation, and oxidative stress to cognitive decline, highlighting the potential role of ferroptosis, a distinct iron-dependent form of cell death associated with lipid peroxidation and impaired iron homeostasis. In addition, genetic factors such as pathogenic copy number variations and their complex gene–gene interactions have been implicated in cognitive and neuropsychiatric phenotypes, providing deeper insight into biological susceptibility. Experimental research also suggests that modulation of intracellular signaling pathways, including MEK–ERK-CREB, may ameliorate learning and memory deficits. Together, these findings provide novel mechanistic and therapeutic insights into postoperative cognitive dysfunction (5–7). Notably, Glumac et al. reported that preoperative low-dose glucocorticoids attenuated inflammation and reduced the incidence and severity of early and late POCD, although routine pathways remain undefined (8, 9). This review examines current evidence on postoperative management of hip fracture in patients with CI, with emphasis on epidemiology, bidirectional pathophysiology, delirium prevention, rehabilitation, outcomes, prognostic factors and evidence gaps (2, 4, 10, 11). Our goal is to provide an evidence-based, clinically relevant guide to improving care and outcomes for patients with hip fracture and coexisting CI.

## Methods

This review aims to systematically synthesize the best available evidence to provide clinical guidance for the postoperative management of older adults patients with hip fractures and CI. The literature search covered the period from 1 January 2018 to 31 October 2025, prioritizing high-level evidence such as meta-analyses, randomized controlled trials, and large-scale cohort studies to establish a reliable foundation for clinical decision-making. Databases searched included PubMed^1^ and Web of Science.^2^ The search strategy combined subject headings with free-text terms, structured as follows: [“Hip Fractures” (MeSH) OR “hip fracture” OR “femoral neck fracture”] AND [“Cognitive Dysfunction” (MeSH) OR “Cognitive Impairment” OR “Dementia” OR “Mild Cognitive Impairment”] AND (“Rehabilitation” OR “Delirium” OR “Perioperative Care” OR “Orthogeriatric” OR “Postoperative Cognitive Dysfunction” OR “Outcomes”). Initial searches imposed no language restrictions; however, only English-language studies were ultimately included. Although both animal and human studies were initially considered, only human studies were retained for the final review due to its clinical guidance purpose.

For the purpose of this review, CI was operationally defined using explicit, study-reported criteria to enhance transparency and reproducibility. We considered participants to have CI if the source study met at least one of the following conditions: Documented pre-fracture clinical diagnosis of dementia, as defined by established diagnostic criteria (e.g., DSM or ICD codes), medical records, or registry-based ascertainment; Mild CI (MCI) diagnosed according to recognized clinical frameworks (e.g., Petersen criteria or equivalent operationalized definitions); Objective cognitive screening thresholds, where CI was identified based on validated instruments, most commonly a Mini-Mental State Examination (MMSE) score <24 or a Montreal Cognitive Assessment (MoCA) score <26, unless alternative cut-offs were explicitly justified by the original study. When studies used other standardized tools (e.g., Mini-Cog, Clinical Dementia Rating, or equivalent brief screening instruments), CI classification was accepted as defined by the authors. Given the heterogeneity in CI definitions across studies—including differences in screening instruments, diagnostic thresholds, and ascertainment methods—no attempt was made to impose a unified classification. Instead, findings were interpreted in the context of the operational definition used in each original study. This approach enabled qualitative weighting of evidence while acknowledging that formal risk-of-bias assessment tools were not applied.

To strengthen clinical interpretability, evidence from included studies was appraised using a design-based hierarchical framework rather than formal GRADE scoring, given the narrative nature of this review. Randomized controlled trials evaluating multidisciplinary or orthogeriatric postoperative interventions were considered high-level evidence, particularly when functional outcomes and postoperative complications were prospectively assessed. Large, well-designed prospective cohort studies with standardized cognitive assessments, longitudinal follow-up, and multivariable adjustment were regarded as moderate-to-high level evidence. This structured appraisal informed the weighting of evidence when formulating clinical recommendations and supports evidence-informed decision-making.

Literature screening was conducted independently by two researchers (HR and HZ), including title and abstract screening as well as full-text review. Disagreements were resolved through discussion with a senior researcher (ZW). Exclusion criteria comprised case reports, research protocols, conference abstracts, purely animal studies, studies with fewer than 50 participants, studies not primarily focused on hip fractures, and studies with less than 1 year of follow-up (when follow-up duration was a critical factor). Additionally, guided by key publications such as “The Complex Multidimensional Relationship between CI and Hip Fracture,” we supplemented the review with high-quality studies published between 2023 and 2025 through targeted PubMed searches and reference tracing. The senior researcher (ZW) approved the final list of the literature. A total of 30 studies were included in the narrative synthesis. Evidence was interpreted using a narrative, design-informed approach rather than formal quantitative weighting: meta-analyses and randomized trials were prioritized when available, followed by well-designed prospective cohorts, while retrospective studies were used as complementary evidence. When findings were inconsistent, greater emphasis was placed on studies with stronger methodology, and differences were interpreted in light of variation in CI definitions, patient populations, and outcome measures. As this was a narrative review rather than a formal systematic review, no formal risk-of-bias assessment or GRADE grading was performed. We instead prioritized studies with rigorous design, clear outcome measures, and strong clinical relevance.

### Pathophysiology and cognitive sequelae

#### CI as a contributor to falls

CI increases the risk of falls through multiple mechanisms. Neuropathological changes in CI, such as Alzheimer-type plaques, neurofibrillary tangles, and cerebrovascular lesions, impair not only memory but also the neural circuits that govern balance, gait, and judgment (12–14). Executive dysfunction, impaired attention, slowed processing speed, and visuospatial deficits further exacerbate fall risk (12–15). Dementia often coexists with motor impairments, such as parkinsonian features in Lewy body dementia or gait apraxia in normal-pressure hydrocephalus, which further compound the risk of falls (14–16). Individuals with CI may also forget or disregard safety precautions (e.g., not using walkers or following safe ambulation practices) and may fail to communicate pain or musculoskeletal problems, increasing their instability (15, 17). In essence, CI diminishes the cognitive and physical reserves necessary to maintain balance and avoid hazards, resulting in a higher likelihood of injurious falls (12–15).

#### Impact of hip fracture and surgery on cognition

**Figure 1:**
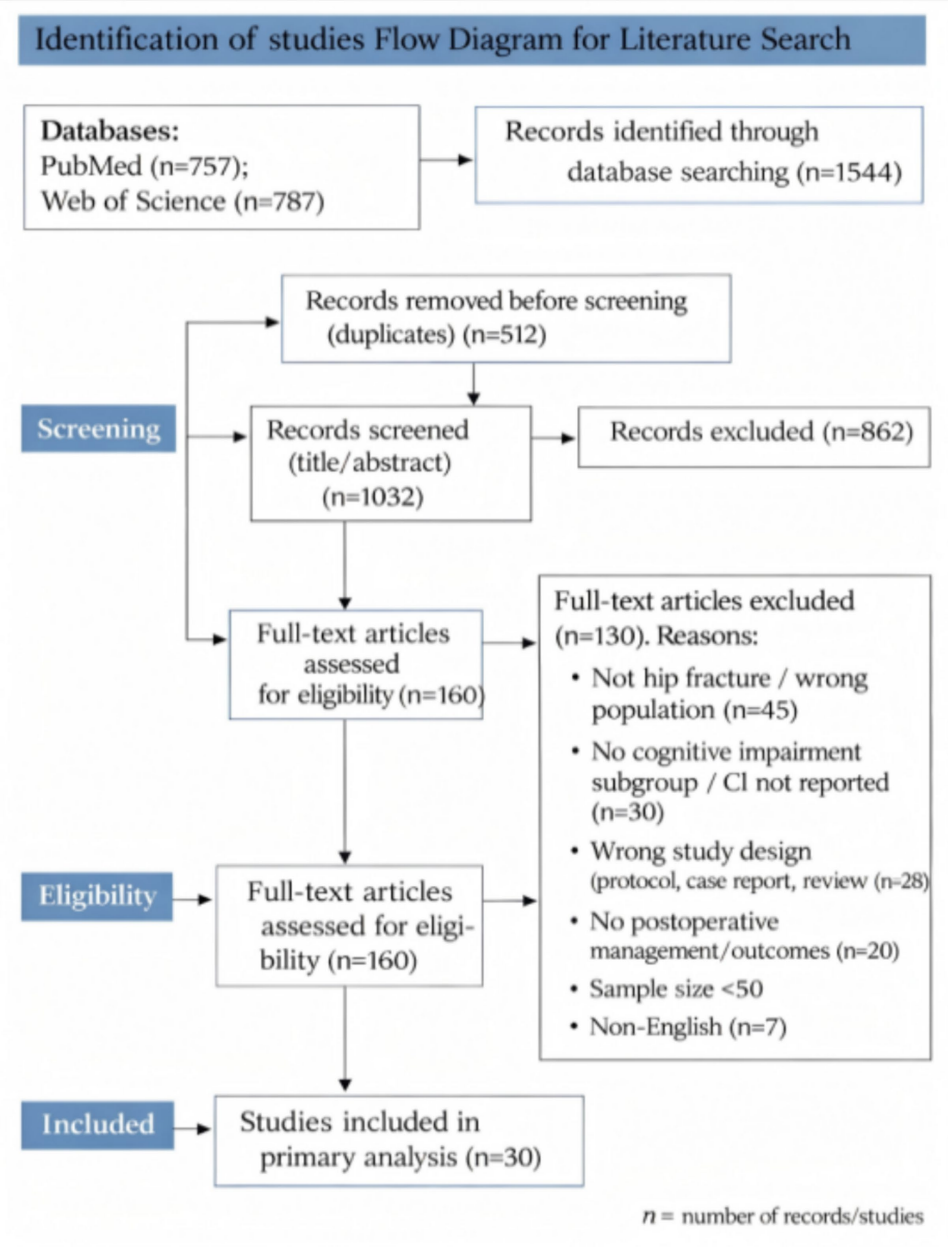
Identification of studies flow diagram for literature search. This figure was generated using Adobe Photoshop 2023 (Adobe Inc., San Jose, CA, United States).

**Figure 2:**
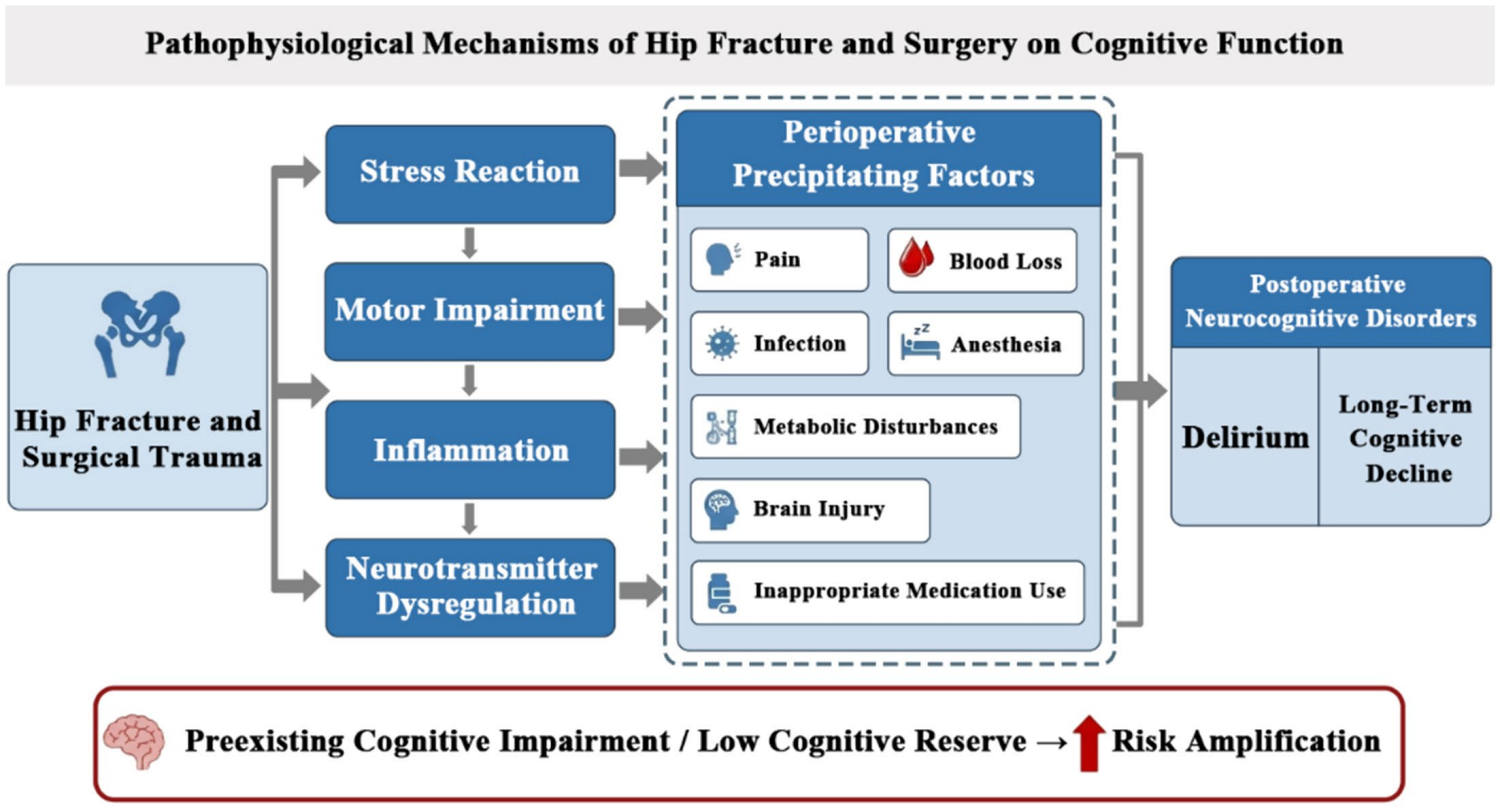
The impact of hip fracture and surgery on cognitive function. This figure was generated using Adobe Photoshop 2023 (Adobe Inc., San Jose, CA, United States).

Hip fracture, surgical interventions, and subsequent hospitalization can significantly impair brain function, particularly in patients with pre-existing cognitive vulnerabilities. Both trauma and surgery are associated with systemic inflammation, active stress responses, and immobility, factors that may detrimentally impact cognitive abilities (18–20). In addition, we systematically summarized the pathophysiological mechanisms through which hip fracture and surgical intervention affect cognitive function (Figure 1). Multiple perioperative factors contribute to the development of postoperative neurocognitive disorders, which may vary from acute delirium to prolonged cognitive decline. Several factors contribute to adverse outcomes in patients, including uncontrolled pain, excessive anesthesia, infection, blood loss, metabolic disturbances, and hemodynamic instability, such as hypotension leading to cerebral hypoperfusion. Additionally, surgical inflammation can induce neuroinflammation and disrupt neurotransmitter function. Other contributing factors include sleep deprivation, sensory disorientation, and polypharmacy, particularly the use of sedatives and anticholinergics (18–21). These factors help explain why patients with pre-existing CI, who often have chronic neuroinflammation or reduced cognitive reserve, are especially vulnerable to postoperative delirium (4, 18, 20). As discussed later, delirium is common after hip fracture and occurs more frequently in patients with dementia or other forms of CI (4, 22). And, the systemic inflammatory response triggered by surgery is a key mechanism underlying postoperative CI and delirium. Studies indicate that preoperative administration of low-dose glucocorticoids effectively suppresses this inflammatory response, with evidence demonstrating a reduced incidence of early postoperative CI in non-orthopedic surgeries. Patients with hip fractures are already in a hyperinflammatory state due to the trauma itself. Existing research, such as Glumac et al., suggest that extending this anti-inflammatory strategy to such patients may hold clinical potential (Figure 2).

**Figure 3:**
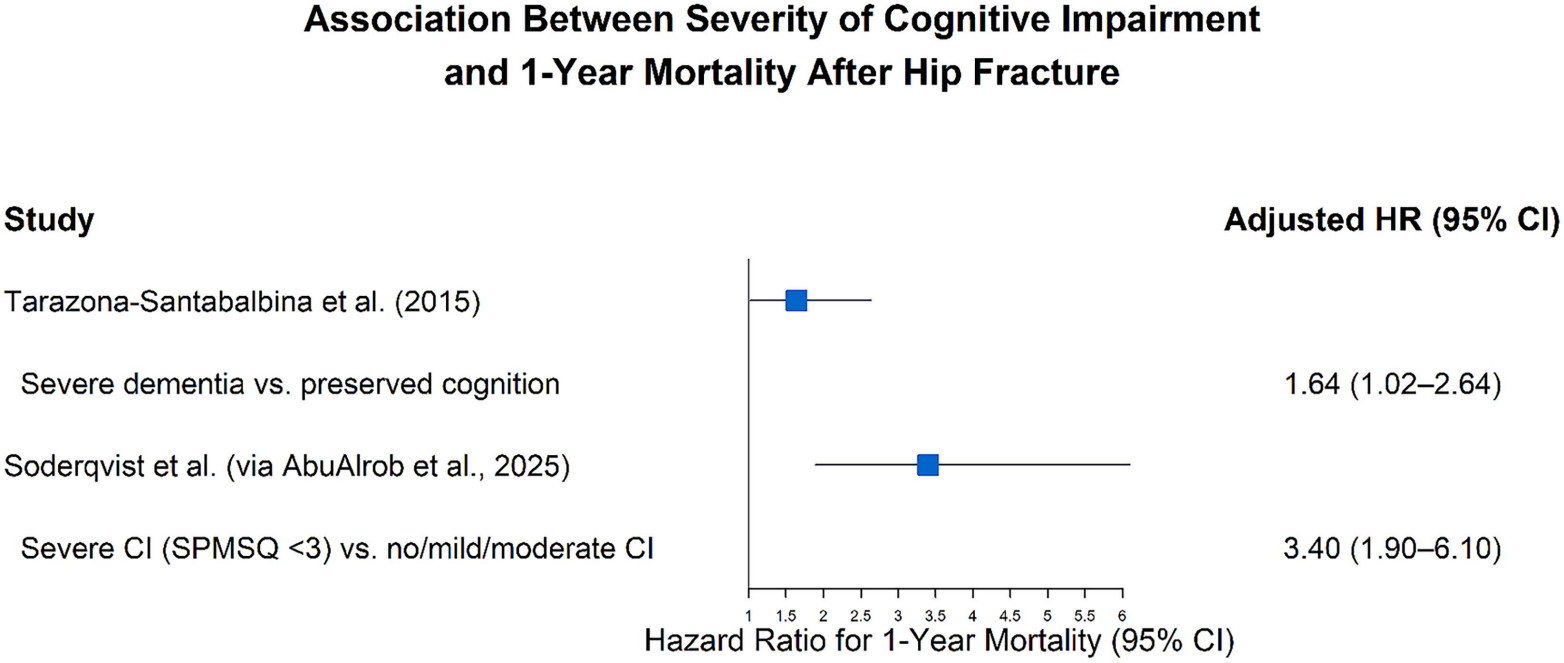
Forest plot illustrating the association between severity of CI and 1-year mortality following hip fracture. Data analysis and visualization for this figure were performed using R version 4.5.2 (R Foundation for Statistical Computing, Vienna, Austria) and RStudio (Posit Software, PBC, Boston, MA, United States).

The severity of CI is a strong independent predictor of poor prognosis after hip fracture. Figure 3 shows the results of the forest plot merging analysis of two representative cohort studies. Adjusted hazard ratios (HR) with 95% confidence intervals from two representative cohort studies are presented for severe CI compared with the reference category (preserved cognition or no/mild/moderate impairment), where an HR > 1 indicates increased mortality risk. The results showed a consistent gradient relationship: the more severe the CI, the higher the 1-year mortality risk (CI group compared with the cognitive function retention group, the corrected risk ratio HR = 1.64, 95% confidence interval 1.02–2.64; compared with the non/mild to moderate CI group, the adjusted hazard ratio HR = 3.40, 95% confidence interval 1.90–6.10). These results emphasize that it is of great clinical significance to carry out routine cognitive screening and develop individualized management strategies for such high-risk groups (23, 24).

### Perioperative and postoperative management

**Figure 4:**
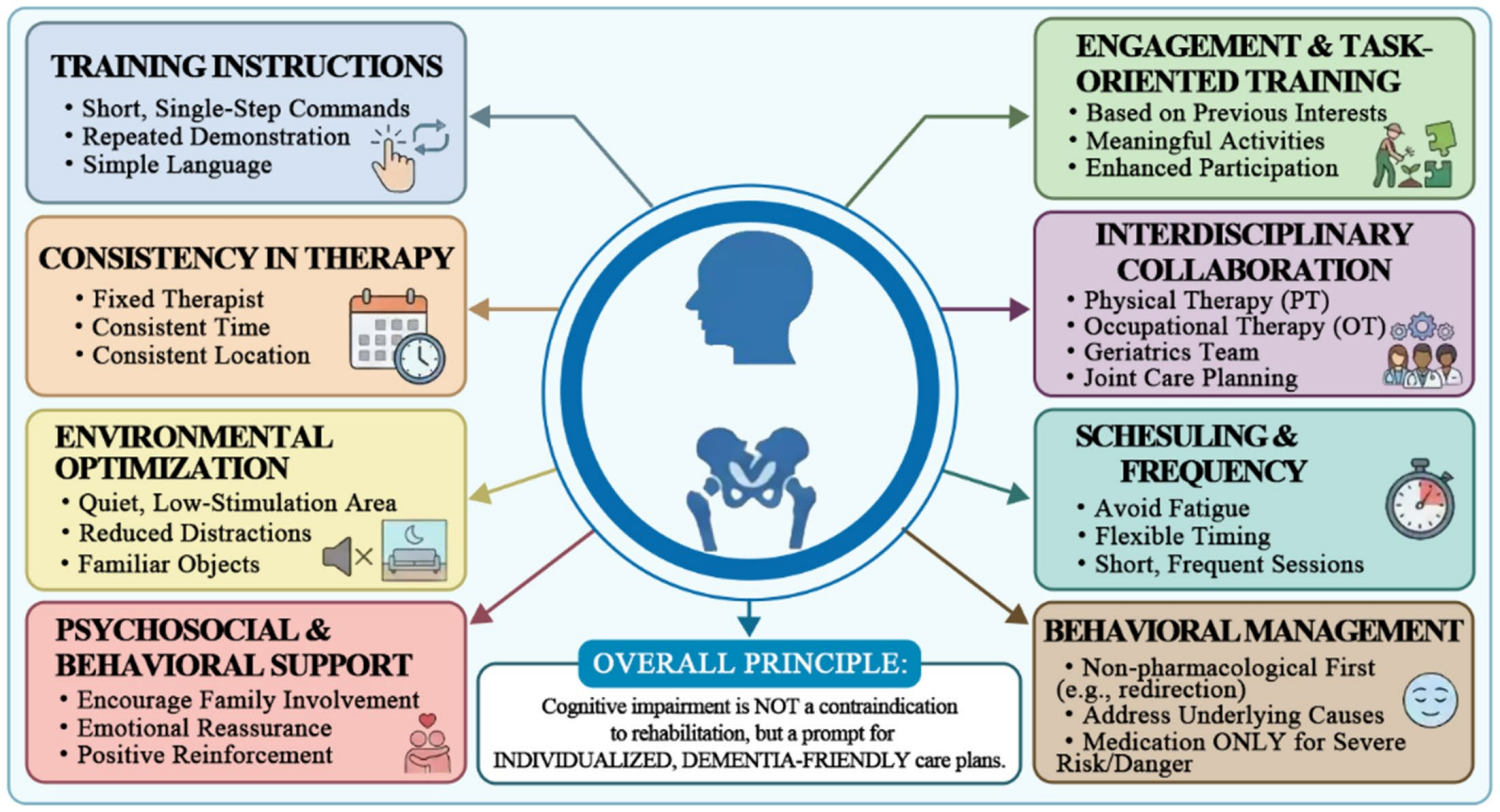
Management strategies for CI after hip fracture surgery. This figure was generated using Adobe Photoshop 2023 (Adobe Inc., San Jose, CA, United States).

Managing hip fractures in patients with CI requires a holistic, multidisciplinary approach that addresses both the fracture itself and the patient’s cognitive vulnerabilities. Traditional hip fracture care pathways, including prompt surgery and early mobilization, must be adapted to meet the needs of patients with CI. Key components of care include geriatric co-management, expedited surgery, prevention and management of delirium, multimodal analgesia, avoidance of deliriogenic medications, early mobilization, and discharge planning tailored to cognitive needs (25, 26) (Figure 3). Detailed rehabilitation strategies are discussed separately in the following section. The following section reviews evidence-based practices across these domains (Figure 4).

#### Orthogeriatric co-management

In the orthogeriatric model, hip fracture care is co-managed by orthopedic surgeons and geriatricians, an approach that is associated with significantly improved patient outcomes. Geriatricians perform comprehensive assessments, optimize medical management by addressing cognition, delirium risk, and nutritional status, and coordinate discharge planning, while orthopedic surgeons focus on fracture repair within shared postoperative goals. A 2022 meta-analysis demonstrated that orthogeriatric care was associated with lower in-hospital and one-year mortality, fewer complications, and shorter lengths of hospital stay compared with standard care(Level 1a) (25). Several studies have also reported a reduced incidence of delirium with co-management, likely reflecting the use of standardized prevention protocols, including daily reorientation, adequate hydration and nutrition, avoidance of deliriogenic medications, and early rehabilitation within orthogeriatric units (26, 27). Geriatric involvement further ensures appropriate assessment of decision-making capacity, engagement of family members in care planning, and management of cognitive- or dementia-related behaviors such as agitation. In a randomized trial, comprehensive geriatric care during hospitalization was associated with improved mobility and recovery of activities of daily living at 4 months, particularly among patients with CI(Level 1b), and showed a trend toward lower one-year mortality compared with standard orthopedic care (11). Best-practice guidelines, such as those from the UK National Hip Fracture Database and the Australian Hip Fracture Standards, recommend geriatric assessment for all patients with fragility hip fractures within 24 h of admission. In summary, orthogeriatric co-management facilitates individualized care for patients with CI by proactively addressing delirium prevention, fall risk, bone health, and discharge planning, and a geriatric specialist or interdisciplinary team should be involved as early as possible.

#### Timing of surgery

Early surgical fixation, ideally within 24 to 48 h of admission, is a cornerstone of improved outcomes (Level 2b) (28, 29). Prompt surgery may help alleviate pain, facilitate earlier mobilization, and is associated with a reduced risk of complications. Surgical delay is associated with increased risks of pneumonia, pressure injuries, prolonged delirium, and mortality (Level 2b) (4, 29–31). For patients with CI, who may be unable to effectively communicate pain or other needs, avoidance of delay is particularly important. While medical stabilization remains essential, unnecessary postponement should be minimized, and comorbid conditions, such as mild cardiac disease, should be rapidly optimized rather than used to justify delay. Each additional 10-h delay has been associated with higher odds of prolonged hospitalization and postoperative complications, ranking second only to delirium as a predictor of adverse outcomes. Fixation within 24 h is also associated with a lower incidence of delirium, likely due to reduced pain and shorter periods of immobilization. Many hospitals have implemented fast-track pathways to expedite surgical care. In addition, minimizing preoperative fasting and discomfort, for example by permitting clear fluids or providing nerve blocks while awaiting surgery, may improve physiological stability. In summary, timely surgery represents one of the most impactful interventions in hip fracture care. When CI is present, clinicians may avoid benign neglect and unwarranted delays. The guiding principle is rapid stabilization without unnecessary postponement, as earlier fixation facilitates earlier mobilization and is associated with a reduced risk of delirium and other complications.

#### Anesthesia and intraoperative management

Regional anesthesia, including spinal or epidural techniques, was previously thought to reduce the risk of delirium compared with general anesthesia; however, recent randomized trials have demonstrated no significant differences in delirium incidence or functional outcomes between contemporary regional and general anesthetic approaches(Level 1b) (32). Consequently, intraoperative priorities should emphasize maintenance of hemodynamic stability and minimization of sedating medications, irrespective of the anesthesia technique used (33). Deep levels of sedation should be avoided, particularly when regional anesthesia is employed, as excessive sedation may negate any potential benefit with respect to delirium risk. The use of processed electroencephalography (EEG) monitoring may assist in preventing excessively deep anesthesia, such as burst suppression, which has been linked to delirium. Short-acting anesthetic agents are preferred, and adequate analgesia, including the use of peripheral nerve blocks, should be provided to attenuate the surgical stress response (34). For patients who are too agitated to tolerate regional anesthesia without significant sedation, general anesthesia remains an appropriate and safer option to ensure procedural safety.

#### Pain management

Effective pain control is crucial, particularly because patients with CI may have difficulty communicating their pain. Poorly controlled pain may precipitate delirium and limit participation in rehabilitation, whereas excessive opioid-related sedation may also contribute to delirium (33). A balanced, multimodal analgesic approach is therefore recommended. This strategy includes early use of regional anesthesia, such as peripheral nerve blocks, scheduled administration of non-opioid analgesics, including acetaminophen, and judicious short-term use of opioids for breakthrough pain (35). Nonsteroidal anti-inflammatory drugs (NSAIDs) may be considered when not contraindicated, although caution is warranted in frail older adults (33). Scheduled acetaminophen in patients with dementia has been shown to reduce opioid requirements and improve overall comfort. Peripheral nerve blocks, such as fascia iliaca compartment blocks or femoral nerve blocks, provide effective analgesia, may reduce the risk of delirium, and decrease opioid-related adverse effects(Level 1a) (34, 36) Ideally, a nerve block should be performed soon after hospital presentation, often in the emergency department, to provide analgesia while awaiting surgery (34). Postoperatively, nerve blocks may be repeated or continued via catheter for 2–3 days. In patients with CI, pain assessment should rely on observational tools, such as the PAINAD scale for advanced dementia, because self-report may be unreliable (37). Analgesics should be administered on a scheduled basis, with careful opioid titration to avoid oversedation, typically starting at lower doses than those used in younger adults. Opioids with fewer neurotoxic metabolites, including hydromorphone or oxycodone, are preferred over agents such as meperidine or high-dose tramadol. Sedating adjuvants medications, including benzodiazepines and gabapentinoids, are generally avoided because of their association with delirium (33). In summary, proactive multimodal pain management addresses a major precipitant of delirium and supports earlier engagement in mobilization and rehabilitation.

#### Delirium prevention and management

Delirium prevention is a top priority in hip fracture patients with CI due to its high incidence and strong association with complications, prolonged hospitalization, functional decline, and increased mortality (38). To clarify priorities throughout the perioperative period, delirium care should be organized as a tiered, stage-based strategy that distinguishes between primary (prevention), secondary (early detection), and tertiary (management) interventions. Primary prevention, which occurs preoperatively and during the immediate perioperative period, should begin upon admission using standardized risk screening tools, such as the 4AT or CAM, to identify high-risk patients, particularly those with dementia, and to initiate a bundled prevention protocol(Level 1a) (39, 40). Core non-pharmacological measures include ensuring access to spectacles and hearing aids; frequent reorientation using clocks, calendars, and verbal cues; sleep protection through daytime light exposure and nighttime noise reduction; early mobilization; and adequate hydration and nutrition, including assisted feeding or intravenous fluids as required (38, 41). Proactive measures should also encompass the prevention and treatment of constipation and urinary retention. Pain management should be optimized through multimodal analgesia, such as paracetamol combined with regional nerve blocks where feasible, while avoiding deliriogenic medications, particularly benzodiazepines, anticholinergics, and excessive opioid sedation. Orthogeriatric co-management may further support adherence to protocols and optimize medical care. Secondary prevention involves postoperative monitoring and early intervention, as the risk of delirium peaks after surgery and may present subtly, including as hypoactive delirium. Routine cognitive assessments using validated tools (4AT/CAM) and structured bedside observations should be implemented (39). At the first signs of cognitive change, it is essential to promptly identify and address common triggers such as uncontrolled pain, urinary retention, fecal impaction or constipation, infection, hypoxia, metabolic disturbances, dehydration, and sleep disruption, while continuing to reinforce the same prevention bundle (35). Tertiary management of established delirium necessitates a prioritization of safety and supportive care (35, 42) This includes maintaining a calm environment, providing reassurance, allowing family presence, or implementing one-to-one observation while avoiding physical restraints. It is essential to systematically address underlying causes. Antipsychotics should be reserved exclusively for cases of severe agitation or psychosis that threaten safety or essential care, administered at the lowest effective dose for the shortest duration, with close monitoring, particularly in patients with dementia (42, 43).

### Rehabilitation strategies for cognitively impaired patients

**Figure 5:**
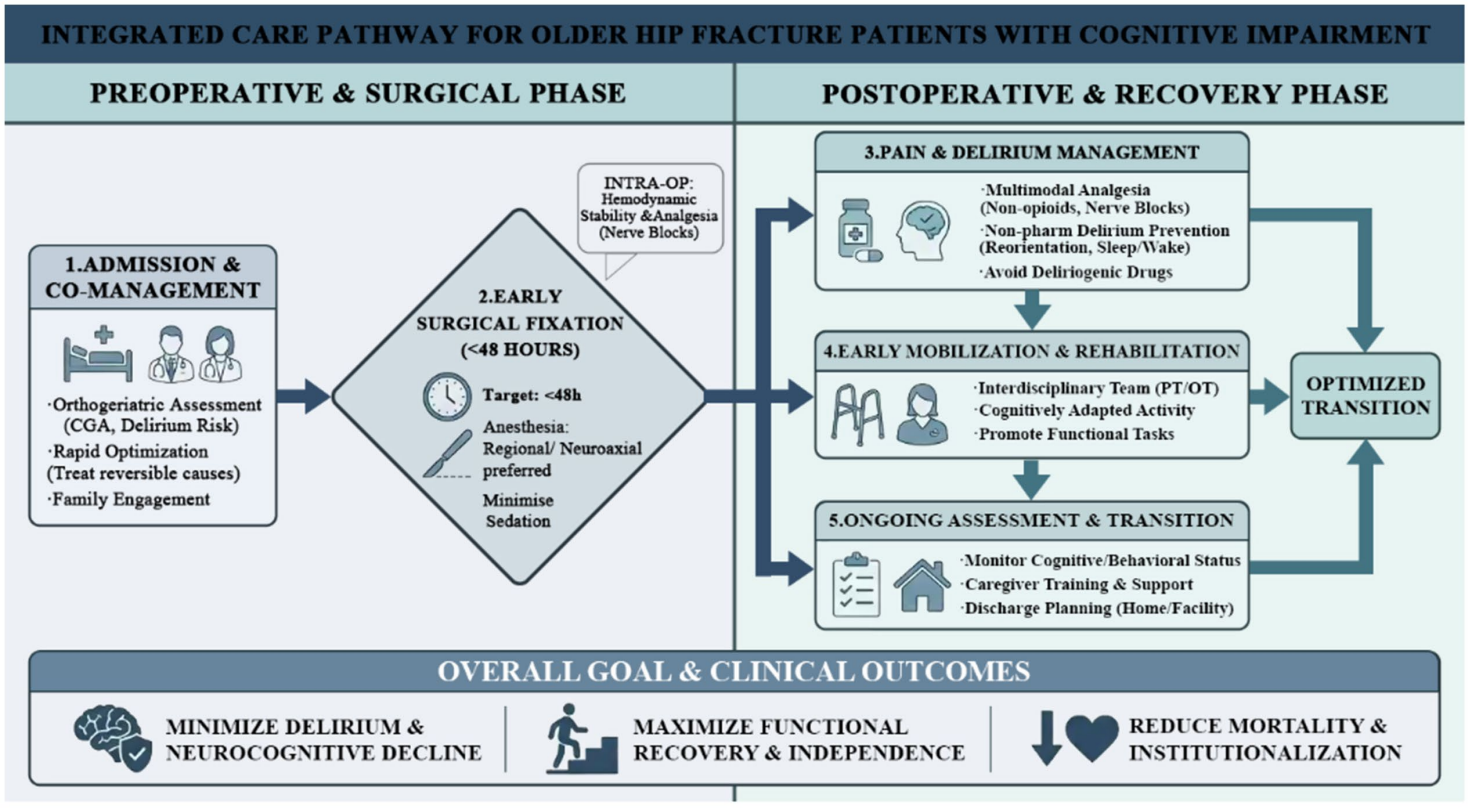
Rehabilitation strategies for patients with hip fractures and CI adaptations to rehabilitation. This figure was generated using Adobe Photoshop 2023 (Adobe Inc., San Jose, CA, United States).

This section focuses on the delivery and adaptation of rehabilitation, whereas longer-term prognosis is addressed in the Outcomes and Prognostic Indicators section. Early, tailored rehabilitation is both feasible and beneficial for patients with CI, yet it has historically been underutilized (44–46). The belief that advanced dementia precludes effective rehabilitation is incorrect, as current evidence does not support withholding therapy (44, 46). A Cochrane review of dementia-specific rehabilitation reported insufficient evidence to draw firm conclusions, largely due to small trial sizes, but suggested that tailored rehabilitation programs may offer potential benefits (44). More recent analyses conclude that, with appropriate modifications, even patients with moderate-to-severe CI may engage in rehabilitation and achieve meaningful functional gains (45). Among patients with CI, rehabilitation is associated with clinically meaningful benefits, including better functional recovery and a greater likelihood of returning to the prior living environment (11, 47, 48). In one randomized trial, patients with dementia who received intensive geriatric rehabilitation after hip fracture showed better recovery of ambulation than those receiving standard care (11, 47, 48). In short, dementia is not a contraindication to therapy; rather, it indicates the need for enhanced, dementia-friendly rehabilitation (44–46) (Figure 5).

Rehabilitation programs for patients with CI require structured modification across content, delivery, and dosing parameters (15, 46, 49). Current guidelines and rehabilitation literature support a standardized but flexible framework, in which the frequency, intensity, and duration of therapy are adjusted according to cognitive severity while maintaining early mobilization and multidisciplinary care (15, 49).

For patients with MCI, rehabilitation may follow conventional post hip fracture protocols, with daily physiotherapy initiated within 24 h postoperatively and sessions lasting 20 to 30 min once or twice daily, provided attention and endurance permit.

For those with moderate CI, shorter but more frequent sessions, such as two 10 to 15 min sessions per day, may be preferable, focusing on task-specific gait training, transfers, and basic activities of daily living, with simplified instructions and frequent repetition (15, 49).

In patients with severe CI, including dementia, rehabilitation should prioritize functional mobility and comfort, using brief five to 10 min, highly structured sessions embedded into routine care, such as assisted walking during toileting or meals, rather than formal exercise blocks (49).

Across all levels of CI, consistency of therapists, predictable schedules, and low-stimulus environments are recommended to reduce confusion and behavioral distress (49). Interdisciplinary input, particularly from occupational therapy, is important to break tasks into manageable steps and adapt activities to cognitive capacity (45, 46). Engagement may be enhanced through meaningful, goal-oriented activities aligned with prior interests (15, 49). Behavioral symptoms should be managed primarily using non-pharmacological strategies, including redirection, rest breaks, adequate analgesia, and environmental optimization, with pharmacological interventions reserved for severe agitation (49). This tiered approach aligns with contemporary hip fracture guidelines and physical therapy consensus, providing a pragmatic framework for tailoring rehabilitation dose and delivery according to cognitive status while preserving access to rehabilitation for patients with CI.

And, this stratified rehabilitation strategy is highly consistent with current hip fracture-related guidelines and evidence-based consensus in the field of physical therapy. By stratifying patients according to cognitive status and delineating distinct priorities in rehabilitation goals, training content, and risk management across subgroups, this framework provides a practical basis for the targeted adjustment of rehabilitation dose, including frequency, intensity, and duration, as well as delivery methods. The framework emphasizes high-intensity, function-oriented structured training in cognitively intact and mildly impaired patients, while prioritizing safety, comfort, and functional maintenance in those with moderate to severe dementia, thereby minimizing risks associated with overtreatment or inappropriate interventions. Collectively, this approach ensures rehabilitation safety while maximizing access to appropriate rehabilitation interventions across different levels of CI (Table 1).

**Table 1:** Stratified rehabilitation framework for geriatric hip fracture patients based on cognitive status.

Cognitive	Rehabilitation framework	Dose parameters (freq/intensity/duration)	Assessment tools	Level of evidence (LoE)
Cognitively Intact	Standard Orthogeriatric Pathway: High-intensity PRT and gait/balance drills	1–2 sessions/day, 70–80% of 1RM intensity, >45 min per session	SPPB, Barthel Index, TUG test	Level 1a
MCI	RCICP: Concurrent physical and cognitive training (Dual-tasking) to prevent cognitive decline	2 sessions/day; Moderate intensity; 30–45 min, focusing on executive function tasks during mobility	MoCA, TMT-A/B	Level 1b
Mild Dementia	Compensatory Functional Training: Use of environmental cues and simplified, repetitive task-oriented exercise	3–4 “micro-sessions”/day, Short duration (15–20 min), Emphasis on habit formation	MMSE, CSHA CFS	Level 2a
Moderate-to-Severe Dementia	Comfort & Safety Protocol: “Errorless Learning” and “Gentle Care” strategies, focus on safe transfers and prevention of disuse syndrome	Frequent short bursts (10 min), Low intensity, Passive/assisted ROM and bed-to-chair mobility	PAINAD (for pain), NPI	Level 2b

Levels of evidence (LoE) were graded according to the Oxford Center for Evidence-Based Medicine (OCEBM) criteria. 1RM, one-repetition maximum; CFS, Clinical Frailty Scale; MCI, mild cognitive impairment; MMSE, Mini-Mental State Examination; MoCA, Montreal Cognitive Assessment; PAINAD, Pain Assessment in Advanced Dementia; PRT, progressive resistance training; RCICP, Rehab-Cognitive Integrated Care; SPPB, Short Physical Performance Battery; TMT-A/B, Trail Making Test Parts A and B; TUG, Timed Up and Go.

#### Rehabilitation settings

The optimal rehabilitation setting for hip fracture patients with CI remains debated. Available options include acute inpatient rehabilitation hospitals, subacute rehabilitation in skilled nursing facilities (SNFs), and extended recovery with therapy in long-term care settings (10, 45). In clinical practice, many patients with dementia are transferred to SNFs (10, 45). Evidence suggests that in patients with mild to moderate dementia, mobility outcomes are comparable between SNF-based and inpatient rehabilitation, whereas severe dementia often precludes eligibility for intensive inpatient programs due to limited capacity for participation. Specialized geriatric rehabilitation units that admit patients with dementia have shown promising results. For example, one trial reported better mobility outcomes in a specialized dementia rehabilitation unit compared to a standard geriatric ward for cognitively impaired patients. Another model that incorporated flexible scheduling, familiar activities, and dementia-trained staff demonstrated better functional outcomes and greater patient engagement compared with standard care (50). When available, such specialized units are preferred for patients with CI, particularly those with dementia (10, 45, 50). Otherwise, close collaboration with SNF therapy teams, often supported by geriatric consultation, is essential to adapt rehabilitation to cognitive and behavioral needs (10, 45). Continuity of care is crucial, as a comprehensive handover from the hospital to the rehabilitation facility, including information on cognitive status, effective strategies, and ongoing goals, facilitates a smoother transition and may support better outcomes (45).

#### Functional goals of rehabilitation

The care team and family should establish realistic yet positive functional goals. Patients with MCI can often regain near pre-fracture levels of ambulation and basic activities of daily living (ADLs). Patients with moderate dementia typically recover the ability to walk with a walker (if previously ambulatory) and may perform personal care with supervision, although they may require more assistance than before. Patients with severe dementia who were marginally ambulatory prior to the fracture may not regain independent walking; however, rehabilitation may still improve transfers, sitting balance, and overall comfort, and prevent complications such as contractures. In all cases, rehabilitation helps prevent deconditioning, pressure ulcers, and muscle atrophy—even when full mobility recovery is unattainable. Families may perceive therapy as futile in advanced dementia; however, even in end-stage disease, individualized exercise programs can temporarily improve or maintain function (51). Additionally, rehabilitation offers psychosocial benefits: structured engagement can reduce agitation and other responsive behaviors driven by boredom or discomfort.

#### Caregiver training and discharge planning

As part of rehabilitation, occupational therapists and social workers should train caregivers in safe assistance techniques for the transition home or to long-term care. Training should include instruction in safe transfer techniques, appropriate use of assistive devices, and home modifications, such as ramps and grab bars, to accommodate both cognitive and mobility limitations (52, 53). Caregivers should also be instructed in home fall-prevention measures, such as removing hazards, securing rugs, and installing grab bars, as a second fracture can be devastating (53, 54). Many hip fracture patients with CI will require long-term supervision or assistance to ambulate safely. Patients who previously lived alone may need a home health aide or family caregiver after discharge, or may need to transition to assisted living or memory care if independent living is unsafe (52, 53). Early involvement of social services facilitates the coordination of support and identification of appropriate placement (52, 53).

### Outcomes and prognostic indicators

Older hip fracture patients with CI generally experience worse outcomes across nearly all domains compared to their cognitively intact peers. In contrast to the preceding section, which focuses on rehabilitation delivery and adaptation, this section summarizes prognosis after hip fracture, including mortality, functional recovery, disability, institutionalization, and prognostic markers. A comprehensive review reported that pre-existing CI, including dementia is associated with higher mortality, poorer functional recovery, lower quality of life, and an increased likelihood of institutionalization after hip fracture (55).

#### Mortality

One-year mortality following hip fracture is higher among patients with dementia, often ranging from approximately 30 to 40%, compared with around 20 to 25% in those without dementia. For example, one analysis found that one-year mortality nearly doubled in the dementia group, at approximately 36% versus 20% (55). A 2025 study found that CI independently conferred approximately 2.1-fold higher odds of one-year mortality after hip fracture (55). Although surgical management provides a survival benefit even in patients with dementia, as nonoperative management is associated with substantially higher mortality, underlying CI nevertheless remains a strong predictor of early mortality, especially within the first 3 to 6 months following fracture (55).

#### Functional outcomes

Even without CI, most older patients with hip fracture do not fully regain their pre-fracture function; only about 40–60% recover their previous walking ability. Among those with dementia, an even smaller proportion regain independent ambulation. In one registry, dementia was associated with a greater decline in walking ability at 4 months: about 30% of patients with dementia returned to their pre-fracture mobility compared with approximately 60% of those without dementia (56). Many patients with dementia who show improvement still require assistive devices or human assistance for mobility. For example, among patients who walked independently outdoors before the fracture, 34% of those with dementia walked independently at 4 months versus 66% without dementia (56). CI also limits rehabilitation potential: memory and executive deficits hinder the learning of compensatory strategies, and delirium (more frequent in CI) can erode functional gains. A national registry reported that patients with CI were less likely to ambulate by postoperative day 1 (34% vs. 58%) and less likely to receive rehabilitation services (35% vs. 64%), contributing to worse functional outcomes (56). CI is also associated with longer times to reach functional milestones (e.g., walking distance, stair climbing), often necessitating extended rehabilitation to maximize recovery (56).

#### Disability and ADLs

Following a hip fracture, many patients experience declines in activities of daily living (ADLs), such as bathing, dressing, and toileting (24, 57). Patients with dementia, who often require assistance with some ADLs at baseline, tend to experience further loss of functional capacity (24, 57). At 1 year post-fracture, cognitively impaired patients had lower functional independence scores and were more likely to require assistance with basic ADLs than cognitively intact peers (2, 24). Even when physically capable, patients with CI may not perform activities safely without supervision. Thus, even with mobility gains, many patients with CI require higher levels of care after fracture, such as transitioning from independent living to assisted living or nursing home care (2, 24).

#### Institutionalization

One of the most striking outcome differences is the rate of new nursing home admission after hip fracture (58). CI is strongly associated with a higher likelihood that an older adult will not return home (58). In a Danish cohort, older adults with dementia had nearly five times higher odds of not returning home within 1 year after fracture. Another registry reported that 46% of previously community-dwelling patients with CI were in residential care 30 days after fracture, compared with 11% of those without CI. Even after adjusting for age and physical health, dementia remains an independent predictor of institutional discharge (58). Within the CI group, dementia severity and pre-fracture living situation are key modifiers: individuals already in assisted living or nursing homes typically remain institutionalized, whereas those from private homes may return if dementia is mild and caregiver support is strong (58). Generally, a patient with mild dementia living with family may return home with additional support, whereas a patient with moderate-to-severe dementia living alone will likely require placement in a nursing facility for safety after fracture, even if physical recovery is adequate (58).

#### Delirium as a prognostic marker

Postoperative delirium independently predicts poorer outcomes beyond baseline dementia, including higher mortality, functional decline, and institutionalization (59–61). Patients who develop delirium exhibit higher rates of mortality and institutionalization, regardless of prior cognitive status (59–61). Delirium serves as a marker of frailty and physiological vulnerability (62). In one study, delirium independently predicted six-month mortality and failure to regain independent ambulation, even after adjustment for age and comorbidities (60). Thus, the occurrence of delirium indicates a need for closer follow-up and, when appropriate, more intensive interventions, such as extended geriatric rehabilitation (61, 62). Conversely, cognitively impaired patients who avoid delirium generally have better recovery trajectories, underscoring the critical importance of delirium prevention (59, 61, 62).

#### Severity of CI

The severity of baseline CI is a key prognostic factor (63, 64). Patients with MCI, like Mini-Mental State Examination [MMSE] scores in the low to mid 20s, generally have substantially better outcomes after hip fracture than patients with advanced dementia (MMSE < 10), including higher rates of survival and functional recovery among those with better cognitive performance (45, 56, 63, 65). In a large Medicare cohort, individuals with moderate to severe dementia had higher mortality rates and were less likely to undergo surgery than those with mild dementia (65). They also experienced poorer functional outcomes and were more frequently discharged to institutional care (65). Studies that stratify outcomes by cognitive test scores show that patients screening positive for probable dementia, such as those with low scores on brief screening tools including the Mini-Cog or MMSE, are less likely to regain independent walking at follow-up than those with only mild impairment (56, 63, 64). In clinical practice, cognitive screening scores aid prognostication: lower scores consistently correlate with greater care needs, higher complication rates, and poorer mobility after hip fracture (45, 63–65).

### Cognitive subtype heterogeneity and implications for personalized care

CI in patients with hip fracture is heterogeneous not only in severity but also in clinical phenotype, which may influence fall mechanisms, vulnerability to delirium, and engagement in rehabilitation. The literature frequently treats CI as a single category, yet cognitive profiles characterized by executive dysfunction, attentional impairment, and visuospatial deficits, which are features highlighted in falls-related pathways, may be particularly relevant to gait safety and postoperative reorientation strategies. Moreover, CI often coexists with motor syndromes, such as parkinsonian features in Lewy body dementia or gait apraxia in normal-pressure hydrocephalus, which can further compound instability and complicate early mobilization goals (i.e., early bed-to-chair transfers, standing, and assisted ambulation in the acute phase). Here, mobilization refers to early postoperative physical activity, whereas rehabilitation denotes the broader, structured recovery process aimed at restoring mobility and activities of daily living. From a management perspective, recognizing subtype-related features supports practical tailoring of care. Patients with prominent attentional fluctuation or visuospatial impairment may require stronger environmental cues, simplified instructions, and consistent staffing to reduce disorientation, whereas those with marked motor impairment may benefit from earlier assistive-device planning and closer supervision during mobilization (as an initial component of rehabilitation). Across phenotypes, avoiding deliriogenic medications, optimizing pain control, and maintaining sleep–wake cycles remain universal, but the emphasis and delivery may differ by cognitive–motor profile. Finally, because postoperative outcome studies rarely report subtype-stratified delirium and rehabilitation results, future work should incorporate cognitive phenotyping to clarify whether delirium prevention bundles and rehabilitation pathways should be further individualized by CI subtype.

## Conclusion

Older adults with hip fractures and CI are a highly vulnerable group with complex care needs. CI—present in up to ~40% of hip fracture patients—is strongly associated with higher risks of delirium, medical complications, functional decline, institutionalization, and mortality. Optimizing outcomes requires an interdisciplinary, patient-centered approach integrating geriatric and orthopedic care. Evidence-based strategies include early geriatric co-management (to reduce mortality, delirium, and length of stay); prompt surgical fixation (ideally ≤24 h) to minimize pain and delirium; careful anesthetic management (avoiding deep sedation and maintaining hemodynamic stability); multimodal analgesia with routine regional nerve blocks (to optimize pain control while limiting opioids); and proactive delirium-prevention bundles (including reorientation, sleep–wake regularity, sensory aids, hydration, and early mobilization). If delirium occurs, clinicians should prioritize correcting reversible triggers and nonpharmacologic management, reserving short-term antipsychotics only for severe agitation that threatens safety. However, the strength of evidence is not uniform across these interventions, with more consistent support for orthogeriatric co-management and delirium prevention than for some specific perioperative and rehabilitation strategies.

Specialized rehabilitation is feasible and beneficial for patients with CI, but typically requires modification. Even with advanced dementia, patients can participate in physical therapy with appropriate cueing and achieve meaningful gains in mobility and self-care. Intensive rehabilitation is associated with lower mortality and better functional outcomes than minimal therapy in this population. Thus, CI should prompt enhanced rehabilitation rather than exclusion. Health systems should provide CI-friendly rehabilitation (e.g., shorter, more frequent sessions; familiar routines; caregiver involvement) and ensure adequate resources (such as longer duration and higher staffing ratios) to support recovery. At the same time, rehabilitation studies remain heterogeneous in intervention intensity, patient selection, and cognitive definitions, limiting certainty regarding the optimal rehabilitation model.

Despite these interventions, patients with CI still experience worse overall outcomes (e.g., lower likelihood of regaining independent living and higher one-year mortality). However, implementing best practices can narrow this gap. Orthogeriatric co-management and delirium prevention are linked to shorter hospital stays and improved survival, while tailored rehabilitation increases the likelihood of regaining mobility. Secondary prevention is critical: all patients should receive osteoporosis evaluation and treatment (calcium/vitamin D and antiresorptives, unless contraindicated), plus fall-prevention interventions to reduce future fracture risk. These measures remain underused in patients with CI, representing a clear opportunity for improvement. Educating family caregivers on safe mobility, home modifications, and medication management helps extend hospital gains into the home environment. Caregiver burden is a significant yet frequently overlooked aspect of hip fracture recovery in patients with CI. Evidence from randomized trials, such as the REACH II intervention, shows that structured, multicomponent support for caregivers, including skills training, psychoeducation, and self-care strategies, can substantially improve caregivers’ health and reduce their burden and distress. In line with NICE dementia guidelines, incorporating caregiver-centered interventions into post-hip fracture care could improve patient outcomes and enhance the sustainability of long-term care (66, 67). Nevertheless, direct hip-fracture-specific evidence for long-term caregiver and functional outcomes remains limited.

CI in hip fracture patients varies in severity and phenotype, influencing treatment response and outcomes. Different cognitive patterns, such as executive, attentional, or visuospatial deficits, may underlie distinct fall mechanisms and affect delirium risk, mobility, and rehabilitation engagement. CI often coexists with motor disorders like Parkinsonism or gait apraxia, further hindering recovery. Recognizing these variations may support more individualized care. Patients with attentional or visuospatial deficits may benefit from clear cues and consistent communication, while those with motor symptoms may need early assistive planning and closer supervision. Core management principles including multimodal analgesia, avoidance of deliriogenic drugs, maintenance of sleep–wake rhythm, and early mobilization remain broadly applicable, but their emphasis may differ across cognitive-motor profiles. This study did not stratify patients by CI subtype, and these considerations are therefore theoretical, highlighting a need for future subtype-specific investigation. A 2025 study indicated that sex may alter the strength of the association between CI and one-year mortality after hip fracture, implying effect modification rather than simple confounding (55). Sex-related differences in frailty burden, comorbidity profiles, inflammatory responses, and muscle reserve could plausibly influence delirium susceptibility and rehabilitation tolerance, yet delirium incidence and functional recovery are rarely reported by sex in CI cohorts (68). These issues highlight important gaps in the literature regarding CI subtype- and sex-specific outcomes. Finally, outcomes are shaped by regional healthcare access and resource availability (69). For example, in high-SDI settings such as Australasia, more robust diagnostic systems, better falls documentation, and stronger perioperative/rehab capacity may support delirium monitoring and rehabilitation delivery, whereas low-SDI settings may face underdiagnosis and limited post-acute resources. In China, a six-hospital survey reported 74.5% of HFAF patients from economically developed urban areas versus 25.5% from less developed rural areas, suggesting urban–rural gaps in service availability. Importantly, while many recommended best practices are resource-intensive, they can be adapted to lower-SDI settings using pragmatic, scalable approaches. Where orthogeriatric co-management, delirium teams, or intensive rehabilitation are limited, feasible adaptations include expedited surgery when possible, structured nonpharmacologic delirium prevention, basic multimodal pain control, early mobilization, and active caregiver involvement. In such contexts, prioritizing high-impact, low-cost interventions may offer substantial benefit even in the absence of specialized multidisciplinary teams. Simplified, task-oriented rehabilitation and task shifting to trained nurses or family caregivers may help sustain recovery in resource-constrained environments. These adaptations support the implementation of core principles of care while acknowledging variability in health system capacity across regions. However, evidence from lower-SDI settings remains comparatively sparse, which may limit the generalizability of some recommendations.

In addition, emerging longitudinal evidence suggests that long-term outcomes after hip fracture are strongly influenced by prefracture cognitive status. A large prospective study with 1-year follow-up demonstrated that prefracture CI severity, rather than postoperative delirium, was associated with sustained functional recovery trajectories, underscoring the need for long-term, cognition-informed follow-up (70, 71). And, unplanned surgery/reoperation is a clinically relevant outcome but was not a prespecified core endpoint of this review; therefore, no definitive conclusion can be drawn regarding its independent association with cognitive impairment. This question remains important and warrants further investigation in future studies. Overall, the current literature consistently identifies CI as a marker of poor prognosis, but remains less definitive regarding the optimal intensity, delivery, and long-term effects of specific interventions.

In summary, addressing both the orthopedic injury and cognitive vulnerabilities can improve outcomes and preserve quality of life for hip fracture patients with CI. As the population ages and more patients present with coexisting CI, including dementia adapting hip-fracture care pathways to incorporate these strategies is essential. Ongoing research and quality-improvement initiatives should refine delirium-prevention bundles, rehabilitation protocols, and post-fracture cognitive care to improve recovery and reduce the disproportionate risks faced by this growing population. This narrative review advances the field by integrating perioperative care, rehabilitation, and caregiver support into a comprehensive framework to optimize outcomes for hip fracture patients with CI. At the same time, the evidence base remains methodologically heterogeneous, and further standardized, high-quality studies are needed to strengthen clinical recommendations.

## Footnotes

1
https://pubmed.ncbi.nlm.nih.gov/

2
https://www.webofscience.com/

## References

[1] Sing CW Lin TC Bartholomew S Bell JS Bennett C Beyene K . Global epidemiology of hip fractures: secular trends in incidence rate, post-fracture treatment, and all-cause mortality. J Bone Miner Res. (2023) 38:1064–75. doi: 10.1002/jbmr.4821, 37118993

[2] Taylor ME Harvey LA Crotty M Harris IA Sherrington C Close JCT. Variation in care and outcomes for people after hip fracture with and without cognitive impairment; results from the Australian and New Zealand hip fracture registry. J Nutr Health Aging. (2024) 28:100030. doi: 10.1016/j.jnha.2023.100030, 38388111 PMC12877269

[3] Fernando E Fraser M Hendriksen J Kim CH Muir-Hunter SW. Risk factors associated with falls in older adults with dementia: a systematic review. Physiother Can. (2017) 69:161–70. doi: 10.3138/ptc.2016-14, 28539696 PMC5435396

[4] Ni Chroinin D Chuan A. Post-operative delirium in the patient with hip fracture: the journey from hospital arrival to discharge. Front Med (Lausanne). (2022) 9:1080253. doi: 10.3389/fmed.2022.1080253, 36507517 PMC9728584

[5] Zhong H Liu H Fu Q. Ferroptosis as a therapeutic target in neurodegenerative diseases: exploring the mechanisms and potential of treating Alzheimer's disease and Parkinson's disease. Protein Pept Lett. (2024) 31:759–72. doi: 10.2174/0109298665333926240927074528, 39513303

[6] Lu YH Chen YJ Lin SJ Hsu TR Niu DM Lin WS. Neurological insights into 16p11.2- and 22q11.2-related disorders: a Mini-review. Curr Genomics. (2025) 26:249–59. doi: 10.2174/0113892029338299241211063307, 41235103 PMC12606657

[7] Chen Y Ma S Huo J Ding S Liu Q Li C . Network pharmacology and bioinformatics of flavonoids from *Scutellaria baicalensis* stems: mitigating Abeta-induced cognitive impairment in rats via the MEK-ERK-CREB pathway. Curr Mol Pharmacol. (2024) 17:e381010. doi: 10.2174/0118761429381010250512060455, 40396317

[8] Glumac S Kardum G Sodic L Supe-Domic D Karanovic N. Effects of dexamethasone on early cognitive decline after cardiac surgery: a randomised controlled trial. Eur J Anaesthesiol. (2017) 34:776–84. doi: 10.1097/EJA.0000000000000647, 28985195

[9] Glumac S Kardum G Sodic L Bulat C Covic I Carev M . Longitudinal assessment of preoperative dexamethasone administration on cognitive function after cardiac surgery: a 4-year follow-up of a randomized controlled trial. BMC Anesthesiol. (2021) 21:129. doi: 10.1186/s12871-021-01348-z, 33892653 PMC8063389

[10] Resnick B Beaupre L McGilton KS Galik E Liu W Neuman MD . Rehabilitation interventions for older individuals with cognitive impairment post-hip fracture: a systematic review. J Am Med Dir Assoc. (2016) 17:200–5. doi: 10.1016/j.jamda.2015.10.004, 26612482 PMC4769900

[11] Prestmo A Hagen G Sletvold O Helbostad JL Thingstad P Taraldsen K . Comprehensive geriatric care for patients with hip fractures: a prospective, randomised, controlled trial. Lancet. (2015) 385:1623–33. doi: 10.1016/S0140-6736(14)62409-0, 25662415

[12] Montero-Odasso M Verghese J Beauchet O Hausdorff JM. Gait and cognition: a complementary approach to understanding brain function and the risk of falling. J Am Geriatr Soc. (2012) 60:2127–36. doi: 10.1111/j.1532-5415.2012.04209.x, 23110433 PMC3498517

[13] Vetrano DL Marengoni A. Rethinking delirium: beyond a syndrome of older age. Lancet Healthy Longev. (2025) 6:100751. doi: 10.1016/j.lanhl.2025.100751, 40754361

[14] Callisaya ML Ayers E Barzilai N Ferrucci L Guralnik JM Lipton RB . Motoric cognitive risk syndrome and falls risk: a multi-center study. J Alzheimer's Dis. (2016) 53:1043–52. doi: 10.3233/JAD-160230, 27340851 PMC5139681

[15] Montero-Odasso M van der Velde N Martin FC Petrovic M Tan MP Ryg J . World guidelines for falls prevention and management for older adults: a global initiative. Age Ageing. (2022) 51:205. doi: 10.1093/ageing/afac205, 36178003 PMC9523684

[16] McKeith IG Boeve BF Dickson DW Halliday G Taylor JP Weintraub D . Diagnosis and management of dementia with Lewy bodies: fourth consensus report of the DLB consortium. Neurology. (2017) 89:88–100. doi: 10.1212/WNL.0000000000004058, 28592453 PMC5496518

[17] Achterberg W Lautenbacher S Husebo B Erdal A Herr K. Pain in dementia. Pain Rep. (2020) 5:e803. doi: 10.1097/PR9.0000000000000803, 32072098 PMC7004504

[18] Marcantonio ER. Delirium in Hospitalized Older Adults. N Engl J Med. (2017) 377:1456–66. doi: 10.1056/NEJMcp1605501, 29020579 PMC5706782

[19] Needham MJ Webb CE Bryden DC. Postoperative cognitive dysfunction and dementia: what we need to know and do. Br J Anaesth. (2017) 119:i115–25. doi: 10.1093/bja/aex354, 29161395

[20] Evered LA Silbert BS. Postoperative cognitive dysfunction and noncardiac surgery. Anesth Analg. (2018) 127:496–505. doi: 10.1213/ANE.0000000000003514, 29889707

[21] Aldecoa C Bettelli G Bilotta F Sanders RD Audisio R Borozdina A . European Society of Anaesthesiology evidence-based and consensus-based guideline on postoperative delirium. Eur J Anaesthesiol. (2017) 34:192–214. doi: 10.1097/EJA.0000000000000594, 28187050

[22] Inouye SK Westendorp RG Saczynski JS. Delirium in elderly people. Lancet. (2014) 383:911–22. doi: 10.1016/S0140-6736(13)60688-1, 23992774 PMC4120864

[23] Tarazona-Santabalbina FJ Belenguer-Varea A Rovira Daudi E Salcedo Mahiques E Cuesta Peredo D Domenech-Pascual JR . Severity of cognitive impairment as a prognostic factor for mortality and functional recovery of geriatric patients with hip fracture. Geriatr Gerontol Int. (2015) 15:289–95. doi: 10.1111/ggi.12271, 25164866

[24] AbuAlrob H Afeef VM Shurman A Shulkin A Azizudin A Hillier L . Scoping review exploring the impact of hip fracture in older adults with cognitive impairment or dementia. BMJ Open. (2025) 15:e093893. doi: 10.1136/bmjopen-2024-093893, 40288797 PMC12035481

[25] Van Heghe A Mordant G Dupont J Dejaeger M Laurent MR Gielen E. Effects of Orthogeriatric care models on outcomes of hip fracture patients: a systematic review and Meta-analysis. Calcif Tissue Int. (2022) 110:162–84. doi: 10.1007/s00223-021-00913-5, 34591127 PMC8784368

[26] Callan KT Donnelly M Lung B McLellan M DiGiovanni R McMaster W . Risk factors for postoperative delirium in orthopaedic hip surgery patients: a database review. BMC Musculoskelet Disord. (2024) 25:71. doi: 10.1186/s12891-024-07174-x, 38233831 PMC10792907

[27] Mant SJ Amadi-Livingstone C Ahmed MH Panourgia M Owles H Pearce O. Orthogeriatric care following hip fracture: improving post-operative outcomes in an aged population. Life. (2024) 14:503. doi: 10.3390/life14040503, 38672773 PMC11050858

[28] Care ACoSaQiH. Hip Fracture Clinical Care Standard. Sydney: Care ACoSaQiH (2023).

[29] Switzer JA O'Connor MI. AAOS Management of hip Fractures in older adults evidence-based clinical practice Guideline. J Am Acad Orthop Surg. (2022) 30:e1297–301. doi: 10.5435/JAAOS-D-22-00273, 36200818

[30] Pincus D Ravi B Wasserstein D Huang A Paterson JM Nathens AB . Association between wait time and 30-day mortality in adults undergoing hip fracture surgery. JAMA. (2017) 318:1994–2003. doi: 10.1001/jama.2017.17606, 29183076 PMC5820694

[31] Warren M Bretherton C Parker M. Delay to surgery beyond 12 hours is associated with increased hip fracture mortality. Eur J Orthop Surg Traumatol. (2024) 34:2973–80. doi: 10.1007/s00590-024-03997-5, 38844565 PMC11377486

[32] Neuman MD Feng R Carson JL Gaskins LJ Dillane D Sessler DI . Spinal anesthesia or general anesthesia for hip surgery in older adults. N Engl J Med. (2021) 385:2025–35. doi: 10.1056/NEJMoa2113514, 34623788

[33] American Geriatrics Society Beers Criteria. American Geriatrics Society 2023 updated AGS beers criteria(R) for potentially inappropriate medication use in older adults. J Am Geriatr Soc. (2023) 71:2052–81. doi: 10.1111/jgs.18372 37139824 PMC12478568

[34] Guay J Kopp S. Peripheral nerve blocks for hip fractures in adults. Cochrane Database Syst Rev. (2020) 2021:CD001159. doi: 10.1002/14651858.CD001159.pub3 PMC8130997 33238043

[35] Lee S Khoujah D Eagles D Kennedy M Lo AX Nickel CH . GRADE-based clinical practice guidelines for emergency department delirium risk stratification, screening, and brain imaging in older patients with suspected delirium. Acad Emerg Med. (2026) 33:e70167. doi: 10.1111/acem.70167, 41146403 PMC12875304

[36] Duan R Cao L Zhang H Li P Wu X Li J. The effect of fascia iliaca compartment block on postoperative delirium in elder adults undergoing hip surgery: a systematic review and meta-analysis of randomized controlled trials. Int J Orthop Trauma Nurs. (2024) 54:101122. doi: 10.1016/j.ijotn.2024.101122, 39047334

[37] Canton-Habas V Carrera-Gonzalez MDP Moreno-Casbas MT Rich-Ruiz M. Spanish adaptation and validation of the pain assessment scale in advanced dementia (PAINAD) in patients with dementia and impaired verbal communication: cross-sectional study. BMJ Open. (2021) 11:e049211. doi: 10.1136/bmjopen-2021-049211, 34158307 PMC8220480

[38] Qi YM Li YJ Zou JH Qiu XD Sun J Rui YF. Risk factors for postoperative delirium in geriatric patients with hip fracture: a systematic review and meta-analysis. Front Aging Neurosci. (2022) 14:960364. doi: 10.3389/fnagi.2022.960364, 35992597 PMC9382199

[39] National Institute for Health and Care Excellence, Guidelines. Delirium: Prevention, Diagnosis and Management in hospital and long-term care. London: National Institute for Health and Care Excellence, Guidelines (2023). 31971702

[40] Tieges Z Maclullich AMJ Anand A Brookes C Cassarino M O'Connor M . Diagnostic accuracy of the 4AT for delirium detection in older adults: systematic review and meta-analysis. Age Ageing. (2021) 50:733–43. doi: 10.1093/ageing/afaa224, 33951145 PMC8099016

[41] Siddiqi N Harrison JK Clegg A Teale EA Young J Taylor J . Interventions for preventing delirium in hospitalised non-ICU patients. Cochrane Database Syst Rev. (2016) 3:CD005563. doi: 10.1002/14651858.CD005563.pub3, 26967259 PMC10431752

[42] Nikooie R Neufeld KJ Oh ES Wilson LM Zhang A Robinson KA . Antipsychotics for treating delirium in hospitalized adults: a systematic review. Ann Intern Med. (2019) 171:485–95. doi: 10.7326/M19-1860, 31476770

[43] Oh ES Needham DM Nikooie R Wilson LM Zhang A Robinson KA . Antipsychotics for preventing delirium in hospitalized adults: a systematic review. Ann Intern Med. (2019) 171:474–84. doi: 10.7326/M19-1859, 31476766

[44] Kamimura T Kobayashi Y Tamaki S Koinuma M. Impact of Prefracture cognitive impairment and postoperative delirium on recovery after hip fracture surgery. J Am Med Dir Assoc. (2024) 25:104961. doi: 10.1016/j.jamda.2024.01.030, 38428834

[45] Cadel L Kuluski K Wodchis WP Thavorn K Guilcher SJT. Rehabilitation interventions for persons with hip fracture and cognitive impairment: a scoping review. PLoS One. (2022) 17:e0273038. doi: 10.1371/journal.pone.0273038, 35969624 PMC9377630

[46] Endersby RVW Fifen JJ Ip VHY. Non-operative management of hip fracture. Anaesthesia. (2025) 81:70042. doi: 10.1111/anae.70042, 41099371

[47] Shyu YI Tsai WC Chen MC Liang J Cheng HS Wu CC . Two-year effects of an interdisciplinary intervention on recovery following hip fracture in older Taiwanese with cognitive impairment. Int J Geriatr Psychiatry. (2012) 27:529–38. doi: 10.1002/gps.2750, 21732418

[48] Shyu YI Tseng MY Liang J Tsai WC Wu CC Cheng HS. Interdisciplinary intervention decreases cognitive impairment for older Taiwanese with hip fracture: 2-year follow-up. Int J Geriatr Psychiatry. (2013) 28:1222–31. doi: 10.1002/gps.3945, 23504666

[49] Pink J O’Brien J Robinson L Longson D Guideline C. Dementia: assessment, management and support: summary of updated NICE guidance. BMJ. (2018) 361:k2438. doi: 10.1136/bmj.k2438, 29925626

[50] McGilton KS Davis A Mahomed N Flannery J Jaglal S Cott C . An inpatient rehabilitation model of care targeting patients with cognitive impairment. BMC Geriatr. (2012) 12:21. doi: 10.1186/1471-2318-12-21, 22631877 PMC3444411

[51] Papamichail P Sagredaki ML Bouzineki C Kanellopoulou S Lyros E Christakou A. The effectiveness of an exercise program on muscle strength and range of motion on upper limbs, functional ability and depression at early stage of dementia. J Clin Med. (2024) 13:136. doi: 10.3390/jcm13144136, 39064174 PMC11278101

[52] Chu CH Paquin K Puts M McGilton KS Babineau J van Wyk PM. Community-based hip fracture rehabilitation interventions for older adults with cognitive impairment: a systematic review. JMIR Rehabil Assist Technol. (2016) 3:e3. doi: 10.2196/rehab.5102, 28582255 PMC5454562

[53] Curtis K Moules P McKenzie J Weidl L Selak T Binks S . Development of an early activation hip fracture care bundle and implementation strategy to improve adherence to the National hip Fracture Clinical Care Standard. J Multidiscip Healthc. (2021) 14:2891–903. doi: 10.2147/JMDH.S323678, 34703242 PMC8524060

[54] Sherrington C Fairhall N Wallbank G Tiedemann A Michaleff ZA Howard K . Exercise for preventing falls in older people living in the community: an abridged Cochrane systematic review. Br J Sports Med. (2020) 54:885–91. doi: 10.1136/bjsports-2019-101512, 31792067

[55] Yang Z Wang YD Zhang BF. Sex modified the association between cognitive impairment and 1-year mortality in older adults with hip fractures. Sci Rep. (2025) 15:18747. doi: 10.1038/s41598-025-03835-6, 40437105 PMC12119921

[56] Linder P Religa DD Gustavsson F Eriksdotter M Hedstrom M Hagg S. Impact of dementia on post-hip fracture walking ability: a stratified analysis based on pre-fracture mobility in Swedish cohorts of older adults. BMC Geriatr. (2024) 24:970. doi: 10.1186/s12877-024-05524-x, 39592980 PMC11590525

[57] Casafont C Gonzalez-Garcia MJ Maranon-Echeverria A Cobo-Sanchez JL Bravo M Piazuelo M . Profile of patients with dementia or cognitive impairment hospitalized with a proximal femur fracture requiring surgery. Int J Environ Res Public Health. (2022) 19:35270492. doi: 10.3390/ijerph19052799, 35270492 PMC8910143

[58] Wahlsten LR Smedegaard L Brorson S Gislason G Palm H. Living settings and cognitive impairment are stronger predictors of nursing home admission after hip fracture surgery than physical comorbidities a nationwide Danish cohort study. Injury. (2020) 51:2289–94. doi: 10.1016/j.injury.2020.06.041, 32622625

[59] Krogseth M Wyller TB Engedal K Juliebo V. Delirium is a risk factor for institutionalization and functional decline in older hip fracture patients. J Psychosom Res. (2014) 76:68–74. doi: 10.1016/j.jpsychores.2013.10.006, 24360144

[60] Bellelli G Mazzola P Morandi A Bruni A Carnevali L Corsi M . Duration of postoperative delirium is an independent predictor of 6-month mortality in older adults after hip fracture. J Am Geriatr Soc. (2014) 62:1335–40. doi: 10.1111/jgs.12885, 24890941

[61] Zhao S Sun T Zhang J Chen X Wang X. Risk factors and prognosis of postoperative delirium in nonagenarians with hip fracture. Sci Rep. (2023) 13:2167. doi: 10.1038/s41598-023-27829-4, 36750657 PMC9905086

[62] Chen Y Liang S Wu H Deng S Wang F Lunzhu C . Postoperative delirium in geriatric patients with hip fractures. Front Aging Neurosci. (2022) 14:1068278. doi: 10.3389/fnagi.2022.1068278, 36620772 PMC9813601

[63] Pislaru AI Sirbu I Albisteanu SM Stefaniu R Turcu AM Grigoras G . Geriatric assessment as an important tool for post-hip surgery prognosis in seniors. Nurs Rep. (2025) 15:40710956. doi: 10.3390/nursrep15070262 PMC12299817 40710956

[64] Sheehan KJ Williamson L Alexander J Filliter C Sobolev B Guy P . Prognostic factors of functional outcome after hip fracture surgery: a systematic review. Age Ageing. (2018) 47:661–70. doi: 10.1093/ageing/afy057, 29668839

[65] Adler RR Xiang L Shah SK Clark CJ Cooper Z Mitchell SL . Hip fracture treatment and outcomes among community-dwelling people living with dementia. JAMA Netw Open. (2024) 7:e2413878. doi: 10.1001/jamanetworkopen.2024.13878, 38814642 PMC11140536

[66] National Institute for Health and Care Excellence, Guidelines. Dementia: Assessment, Management and Support for People living with Dementia and their Carers. London: National Institute for Health and Care Excellence, Guidelines (2018). 30011160

[67] Elliott AF Burgio LD Decoster J. Enhancing caregiver health: findings from the resources for enhancing Alzheimer's caregiver health II intervention. J Am Geriatr Soc. (2010) 58:30–7. doi: 10.1111/j.1532-5415.2009.02631.x, 20122038 PMC2819276

[68] Dong X Zhang X Hu F Yang S Hong Z Geng Q. Association of frailty with adverse outcomes in surgically treated geriatric patients with hip fracture: a meta-analysis and trial sequential analysis. PLoS One. (2024) 19:e0305706. doi: 10.1371/journal.pone.0305706, 38905251 PMC11192356

[69] Zhang B Dou B Li K. Global, regional, and national burden of hip fractures attributable to falls in older adults: changes from 1990-2021 and 2036 projections. Front Public Health. (2025) 13:1674881. doi: 10.3389/fpubh.2025.1674881, 41048275 PMC12488404

[70] Birkner D Pigorsch M Riedlinger D Mockel M Lindner T Schenk L . The vulnerability of hip fracture patients with cognitive impairment: an analysis of health conditions, hospital care, and outcomes. BMC Geriatr. (2025) 25:99. doi: 10.1186/s12877-025-05744-9, 39953428 PMC11829398

[71] Lim SK Lim JY. Hip fracture and cognitive impairment in older adults-integrated approaches to rehabilitation: a narrative review. Ewha Med J. (2025) 48:e59. doi: 10.12771/emj.2025.00801, 41223888 PMC12611440

